# Ethnic disparities in postpartum hemorrhage after cesarean delivery: a retrospective case–control study

**DOI:** 10.1007/s00540-021-02899-8

**Published:** 2021-01-28

**Authors:** Yu Wang, Hexin Gao, Tuvshin Bao, Lijuan Yang, Guifeng Ding, Demu Ba, Shujun Sun, Yun Lin, Shanglong Yao

**Affiliations:** 1 Department of Anesthesiology, Union Hospital, Tongji Medical College, Huazhong University of Science and Technology, Wuhan, China; 2 Department of Anesthesiology, Maternal and Child Health Hospital of Xinjiang Uyghur Autonomous Region, Urumqi, Xinjiang Uygur Autonomous Region China; 3 Department of Anesthesiology, Inner Mongolia Maternal and Child Health Care Hospital, Hohhot, Inner Mongolia Autonomous Region China; 4 Department of Anesthesiology, People’s Hospital of Bozhou, Bole, Xinjiang Uygur Autonomous Region China

**Keywords:** Postpartum hemorrhage, Ethnicity, Obstetric anesthesia

## Abstract

**Purpose:**

To explore the relationship of ethnicity and postpartum hemorrhage (PPH) for women who underwent cesarean delivery (CD) and examine the risk factors for PPH in distinct ethnic groups in China.

**Methods:**

We conducted case–control studies with the maternity data from the 11,778 CD cases, in Xinjiang Uygur Autonomous Region. Initially, multivariable logistic regression was used to estimate the disparity of race-ethnicity on the risk of PPH in ethnic Han, Uygur, Hui and Kazakh. Then, we performed case–control studies within two major ethnic groups, identifying the specific risk factors for PPH.

**Results:**

Ethnic Uygur were associated with a statistically significant increased odds [adjusted odds ratios (aOR) 2.05; 95% confidence interval (CI) 1.26–3.33] of PPH compared with ethnic Han. For subgroup analyses, in Uygur subgroup, general anesthesia (aOR 7.78; 95% CI 2.31–26.20); placenta previa (aOR 11.18; 95% CI 3.09–40.45); prenatal anemia (aOR 4.84; 95% CI 2.44–9.60); emergency surgery (aOR 4.22; 95% CI 1.95–9.13) were independently associated with PPH. In Han subgroup, general anesthesia (aOR 5.70; 95% CI 1.89–17.26); placenta previa (aOR 20.08; 95% CI 6.35–63.46); multiple pregnancy (aOR 7.21; 95% CI 1.61–32.37); body mass index (aOR 1.19; 95% CI 1.07–1.31) were the risk factors to PPH.

**Conclusion:**

Uygur have more tendency to PPH compared to Han, and risk factors for PPH in Uygur and Han groups may differ. Knowing these differences may be meaningful when planning interventions and resources for high-risk patients undergoing cesarean delivery, and we need more research aimed at risk factors for PPH.

## Introduction

Postpartum hemorrhage (PPH) is a severe complication that may result in adverse outcomes of perinatal mothers and infants. Worldwide, a woman dies due to massive PPH approximately every 4 min [1]. PPH is the major cause of maternal mortality in China which accounted for 28% of all maternal deaths in 2013 [2]. It is also an important cause of pregnancy-related morbidity, such as multi-organ failure and peripartum hysterectomy [1, 3]. There are a variety of etiologies of PPH, including uterine atony, retained placenta, lacerations of the birth canal, uterine rupture, placenta accreta, various types of coagulopathies, uterine inversion and infection, each of which has diverse risk factors [4, 5]. The major cause of PPH is uterine atony, accounting for approximately 80% of PPH cases, and often occurs in the absence of recognized risk factors [6]. A number of changes after implementation of the two-child policy in 2016 in obstetric practice and maternal demographics in China may have contributed to an increased rate of PPH, including an increasing rate of cesarean delivery (CD) and more pregnant women of advanced maternal age [7]. It is therefore necessary to determine risk factors for PPH to plan interventions and resources for high-risk patients.

Racial-ethnic disparities are persistent problems in pregnant women's health and obstetric outcomes. In some Western countries, race as an independent risk factor for PPH has been documented [8, 9]. Some retrospective studies indicated racial and ethnic disparities in PPH in the US [8, 9]. CD increases blood loss at delivery and thus is a risk factor of PPH [5]. However, few previous study did pay specific attention to women who underwent CD. Moreover, to date, there are no studies assessing the racial-ethnic disparities in PPH among ethnic groups in China, because of the limited data of PPH among different ethnic groups in the same setting and the same period. Xinjiang Uygur Autonomous Region (referred to as Xinjiang in the remainder of this study) has a large multi-ethnic population with 47 ethnic components, and Urumqi is the capital city of Xinjiang. The ethnic group with the largest population is ethnic Han, the other ethnic groups mainly include ethnic Uygur, Hui and Kazakh. A unique data set from different ethnic groups in Xinjiang Region, accumulates large amounts of cases undergoing CD and suffering from PPH to examine risk factors.

In this study, we conducted a retrospective case–control study to test the hypothesis that the ethnicity is an independent risk factor for PPH. It was further determined whether the individual risk factors for PPH vary among women who underwent CD in the two largest ethnicity subgroups.

## Methods

### Ethics approval and consent to participate

After obtaining Ethics Committee of the Xinjiang Uygur Autonomous Region Maternal and Child Health Hospital approval on 31 January 2018 (Ethical approval number: XJFYLL2018001), we registered our project at the Chinese clinical trial registry (Registration number: ChiCTR1800014752). We had no access to information that could identify individual participants, so our research does not involve patients' personal information and written informed consent was waived.

### Study designs

We performed retrospective analyses with case–control study design to explore risk factors for PPH among ethnic Han, Uygur, Hui and Kazakh in Xinjiang Region. The cases were the CD women diagnosed as PPH and the controls were identified from the CD parturients without PPH selected with matched factors, and the ratio of the case:control was 1:2. Maternal age and gestational weeks were considered as the matching factors because advanced maternal age (age > 35) [10, 11] and preterm births [12] are known risk factors. Premature labor was defined when gestational weeks < 37 compared with reference group defined as gestational weeks ≥ 37.

### Data resources

Data were collected from parturients who underwent CD in the Xinjiang Uygur Autonomous Region Maternal and Child Health Hospital, from January 2014 to January 2017. We extracted information from Electronic Medical Record, Laboratory Information System, Picture Archiving and Communication Systems, and Anesthesia Information System. For each parturient, the following information is extracted and classified: (1) demographic characteristics; (2) obstetric characteristics; (3) comorbidities; (4) fetal conditions; (5) clinical managements. The diagnoses and procedures were recorded using International Classification of Diseases 9th Revision, Clinical Modification codes (ICD-9-CM codes) [13].

### Inclusion criteria and exclusion criteria

Our study only screened women who underwent CD. We included the parturients of ethnic Han, Uygur, Hui and Kazakh, because these are the major ethnic groups that make up the largest proportion. The inclusion criteria were the women who underwent CD; women aged 16 years and older; patients categorized as American Society of Anesthesiologists (ASA) I–III; information of the hospital discharge records was detailed. After the first-round screening, we excluded women from the study for the following reasons: the patients with uncertain or missing hospital records (such as predelivery hemoglobin undetermined or past medical history unrecorded); patients with antenatal bleeding or bleeding owing to preoperative thrombocytopenia and coagulation factor decreased; patients categorized as ASA IV; patients who converted from spinal anesthesia to general anesthesia.

### Primary outcomes and potential confounders

The primary outcome was PPH defined as an estimated blood loss (EBL) ≥ 1000 mL after CD according to ICD-9-CM codes, or red blood cell (RBC) transfusion, both within 24 h [14]. The blood loss was measured based on the amount of fluid in the aspirator and the weight of the dressings, as well as estimated by the surgeons (subjective estimated blood loss). ICD-9-CM codes for PPH are well-verified, showing a positive predictive value of 80% [15]. As blood loss may often be underestimated by clinicians [16, 17], RBC transfusion is a component in the definition of severe PPH [18]. The primary independent variable for this study was ethnicity. We classified ethnicities into four groups: Han, Uygur, Hui and Kazakh, with Han as the reference group. Previous studies [6, 8, 9, 19–24] indicated potential confounders and we divided those variables into 5 categories: (1) demographic characteristics; (2) obstetric characteristics; (3) comorbidities; (4) fetal conditions; (5) clinical managements. The demographic characteristics contained maternal age [19], racial-ethnicity [8, 9], body mass index (BMI) [20, 21], maternal educational level. Gestational weeks, gravidity, parity, number of previous CD, and previous uterine scar were categorized as obstetric characteristics. Relevant maternal comorbidities and fetal conditions were identified using corresponding ICD-9-CM codes and included diabetes, hypertension, gynecological tumor, prenatal anemia, placental abruption, placenta previa, stillbirth, multiple pregnancy, cephalopelvic disproportion, malpresentation, and macrosomia. Clinical managements contained emergency surgery, number of antenatal visits, assisted conception, intrapartum CD, cesarean delivery on maternal request (CDMR), anesthesia methods [23, 24] included general anesthesia and spinal anesthesia.

### Statistical methods

In the primary analysis, we identified women with PPH and matched controls among the four ethnic populations. The univariate analyses were performed to generate crude odds ratios (ORs) with 95% confidence intervals (95% CI). We then fitted multivariable logistic regression models to determine the relationship between the ethnicity and the PPH to generate adjusted ORs (aOR), with adjustments for all variables that *P* value < 0.05 in the univariate analyses. In the stratified analyses, we performed separate analyses among women in ethnic Han and Uygur undergoing CD, with univariable and multivariable logistic regression models.

Before each logistic regression model, restricted cubic spline functions were used to test whether the associations met the assumptions of a linear relationship for each continuous variable associated with PPH. We categorized continuous variables if they were not linearly related to PPH or they could be categorized by clinically relevant cut points (pre-delivery hemoglobin was categorized and cut by 100 g/L to represent the prenatal anemia). Gravidity, parity, number of previous CD, number of antenatal visits, maternal educational level and BMI were regarded as continuous variables. The effect of potential collinearity on the estimates for ethnicity was assessed by calculating a variance inflation factor (VIF) between candidate variables. Collinearity was determined to be insignificant if VIF scores < 10. The Hosmer–Lemeshow statistics were calculated for each model to evaluate the goodness of models’ fit. *P* value < 0.05 was considered statistically significant and all tests were two-tailed. Demographic and clinical characteristics were presented by frequency, proportion, mean, and standard deviation. All statistical analyses were carried out using the SAS 9.4 (SAS Institute, Cary, NC, USA).

## Results

**Fig. 1:**
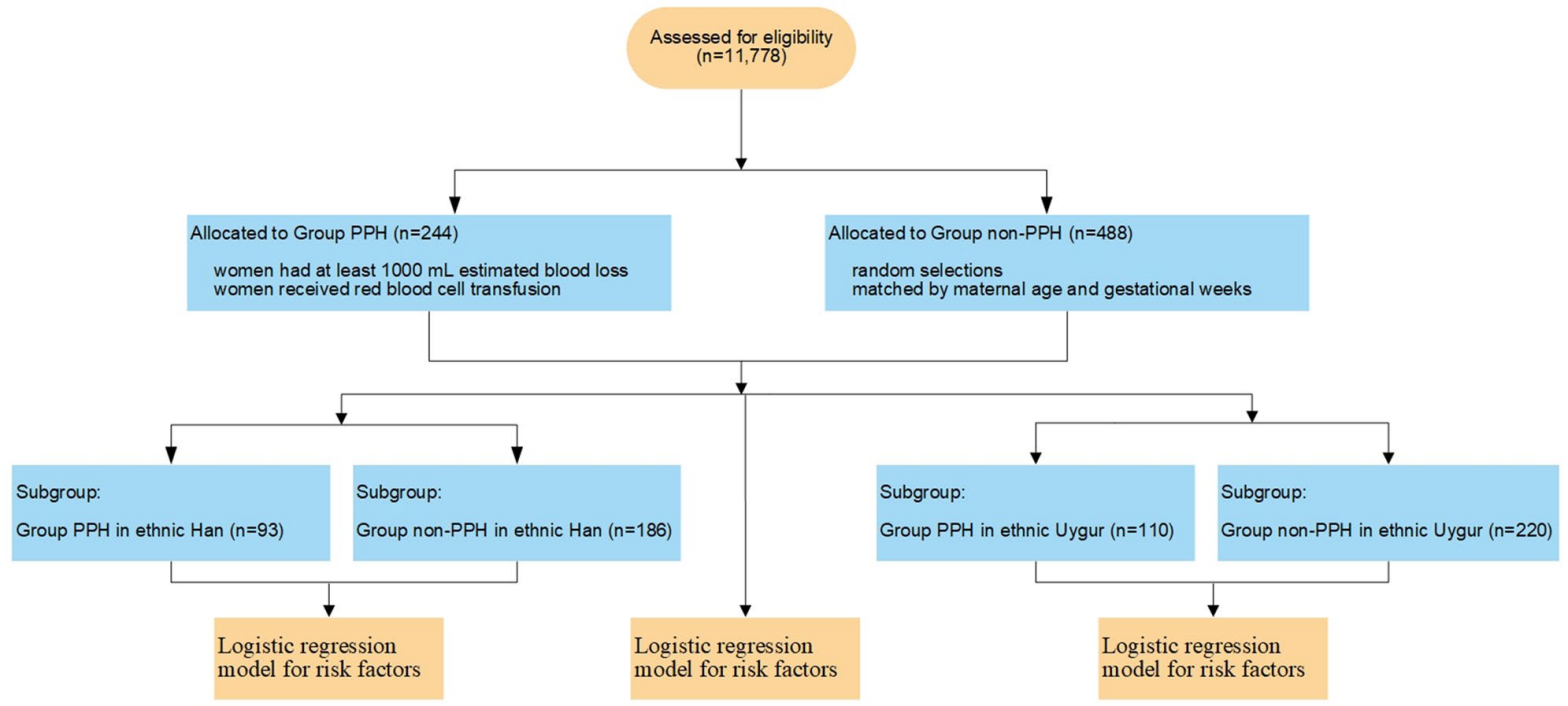
Subject enrollment and analysis flowchart

A total of 11,778 CD cases were included in our study. Most pregnant women were ethnic Han women (43.7%) and Uygur women (39.2%), followed by Hui (9.8%) and Kazakh (7.3%) women. There were 244 cases identified as PPH finally. Among PPH cases, 166 (68.0%) women had at least 1000 mL EBL, and 147 (60.2%) women received RBC transfusion intraoperatively or within 24 h after CD. The overall rate of PPH was 2.1% (95% CI 1.8–2.3%). Subject enrollment and analysis are illustrated in Fig. 1.

In the primary analysis of the four ethnic populations, we identified 244 women with PPH and 488 matched controls. Characteristics of cases and controls were presented in Table 1. Of the 24 potential confounders in Table 1, there were 14 variables with *P* value < 0.05 in the univariate analyses, included ethnicity, general anesthesia, placenta previa, placental abruption, multiple pregnancy, previous uterine scar, gravidity, number of previous CD, number of antenatal visits, maternal educational level, diabetes, still birth, prenatal anemia, emergency surgery. Unadjusted and adjusted odds ratios (aOR) for ethnicities in multivariable analysis were showed in Table 2. After the adjustment for potential mediators, ethnic Uygur and Kazakh were independently associated with PPH, with a statistically significant increased odds of PPH (aOR 2.05; 95% CI 1.26–3.33) and (aOR 3.83; 95% CI 1.80–8.16) in comparison with Han. The *P* value for Hosmer–Lemeshow test was 0.07 > 0.05, indicating good fit of the model.

**Table 1:** Characteristics of included parturients with or without postpartum hemorrhage

Independent variables	PPH (244)	Control (488)
Race/ethnicity		
Han	93 (38.1%)	247 (50.6%)
Uygur	110 (45.1%)	147 (30.1%)
Hui	18 (7.4%)	60 (12.3%)
Kazakh	23 (9.4%)	34 (7.0%)
BMI (weight/height^2^)	29.0 ± 3.6	28.3 ± 3.5
Maternal educational level	2.4 ± 0.7	2.6 ± 0.6
Gravidity	2.3 ± 0.8	1.9 ± 0.8
Parity	1.9 ± 0.7	1.7 ± 0.7
Times of CD	1.6 ± 0.7	1.3 ± 0.5
Scared uterus	131 (53.7%)	159 (32.6%)
Diabetes	41 (16.8%)	41 (8.4%)
Hypertension	12 (4.9%)	10 (2.0%)
Gynecological tumor^1^	6 (2.5%)	9 (1.8%)
Prenatal aknemia	95 (38.9%)	117 (24.0%)
Placental abruption	21 (8.6%)	7 (1.4%)
Placenta previa	73 (29.9%)	17 (3.5%)
Multiple pregnancy	17 (7.0%)	4 (0.8%)
Cephalopelvic disproportion	2 (0.8%)	4 (0.8%)
Macrosomia	112 (45.9%)	213 (43.6%)
Malpresentation^2^	28 (11.5%)	73 (15.0%)
Still birth	15 (6.2%)	5 (1.0%)
Emergency surgery	163 (66.8%)	208 (42.6%)
Number of antenatal visits	3.6 ± 3.9	5.8 ± 4.3
Assisted conception	5 (2.1%)	11 (2.3%)
Intrapartum CD	27 (11.1%)	68 (13.9%)
CDMR	4 (1.6%)	25 (5.1%)
Anesthesia method		
Spinal anesthesia	189 (77.5%)	470 (96.3%)
General anesthesia	55 (22.5%)	18 (3.7%)

Data presented as *n* (%); mean ± SD

*PPH* postpartum hemorrage, *BMI* body mass index, *CD* cesarean delivery, *CDMR* cesarean delivery on maternal request

^1^Gynecological tumor: such as pregnancy with cervical cancer, giant cervical fibroids, subuterine fibroids, etc.

^2^Malpresentation: fetal transverse position, first-born full-term single breech (estimated fetal birth weight > 3500 g) and foot first exposure

**Table 2:** Unadjusted and adjusted odds ratios for ethnicity associated with postpartum hemorrhage

Race/ethnicity	Unadjusted Odds Ratio^1^	Adjusted Odds Ratio^2^	*P* value^3^
Han	Reference	Reference	Reference
Uygur	1.99 (1.41–2.80)	2.05 (1.26–3.33)	0.004
Hui	0.80 (0.45–1.42)	1.27 (0.61–2.65)	0.520
Kazakh	1.80 (1.01–3.21)	3.83 (1.80–8.16)	0.001

^1^A total of 244 cases of postpartum hemorrhage in four ethnic groups were identified, and 488 matched controls. Crude odds ratios (ORs) of ethnicity related to postpartum hemorrhage were calculated, with their 95% confidence intervals (95% CI)

^2^Adjusted odds ratios (ORs) of ethnicity related to postpartum hemorrhage were calculated, with their 95% confidence intervals (95% CI) by multivariable logistic regression included all variables with *P* <  0.05 in the univariable analyses

^3^The *P* value represents the statistical significance of ethnicity in the multivariable model

In stratified analyses, we identified 110 cases in ethnic Uygur and 93 cases in ethnic Han. The matched controls were selected randomly in the Han and Uygur parturients, respectively, with the ratio of the case:control = 1:2. Characteristics of women who are Han or Uygur with and without PPH were presented in Table 3.

**Table 3:** Characteristics in parturients of ethnic Han and Uygur with or without postpartum hemorrhage

Independent variables	Han		Uygur	
	PPH (*n* = 93)	Control (*n* = 186)	PPH (*n* = 110)	Control (*n* = 220)
BMI (weight/height^2^)	29.5 ± 3.6	28.2 ± 3.1	28.8 ± 3.6	29.7 ± 4.5
Maternal educational level	2.5 ± 0.6	2.6 ± 0.6	2.3 ± 0.7	2.6 ± 0.6
Gravidity	2.2 ± 0.8	1.8 ± 0.8	2.4 ± 0.7	1.8 ± 0.8
Parity	1.6 ± 0.7	1.4 ± 0.6	2.2 ± 0.7	1.5 ± 0.7
Number of previous CD	1.5 ± 0.6	1.2 ± 0.5	1.8 ± 0.7	1.3 ± 0.6
Previous uterine scar	43 (46.2%)	44 (23.7%)	71 (64.5%)	63 (28.6%)
Diabetes	21 (22.6%)	17 (9.1%)	14 (12.7%)	25 (11.4%)
Hypertension	5 (5.4%)	3 (1.6%)	6 (5.5%)	10 (4.5%)
Gynecological tumor^1^	4 (4.3%)	2(1.1%)	0	2 (0.9%)
Prenatal anemia	18 (19.4%)	29 (15.6%)	60 (54.5%)	33 (15.0%)
Placental abruption	4 (4.3%)	3 (1.6%)	13 (11.8%)	4 (1.8%)
Placenta previa	29 (31.2%)	5 (2.7%)	32 (29.1%)	5 (2.3%)
Multiple pregnancy	8 (8.6%)	4 (2.2%)	9 (8.2%)	13 (5.9%)
Cephalopelvic disproportion	1 (1.1%)	1 (0.5%)	1 (0.9%)	2 (0.9%)
Macrosomia	23 (24.7%)	27 (14.5%)	16 (14.5%)	38 (17.3%)
Malpresentation^2^	10 (10.8%)	24 (12.9%)	14 (12.7%)	33 (15%)
Stillbirth	3 (3.2%)	0	8 (7.3%)	1 (0.5%)
Emergency surgery	58 (62.4%)	130 (69.9%)	87 (79.1%)	118 (53.6%)
Number of antenatal visits	4.3 ± 4.0	6.3 ± 4.3	2.6 ± 3.2	4.0 ± 3.8
Assisted conception	5 (5.4%)	2 (1.1%)	0	3 (1.4%)
Intrapartum CD	13 (14.0%)	27 (14.5%)	7 (6.4%)	34 (15.5%)
CDMR	1 (1.1%)	5 (2.7%)	2 (1.8%)	4 (1.8%)
Anesthesia method				
Spinal anesthesia	79 (84.9%)	177 (95.2%)	77 (70.0%)	214 (97.3%)
General anesthesia	14 (15.1%)	9 (4.8%)	33 (30.0%)	6 (2.7%)

Data presented as *n* (%); mean ± SD

*PPH* postpartum hemorrage, *BMI* body mass index, *CD* cesarean delivery, *CDMR* cesarean delivery on maternal request

^1^Gynecological tumor: such as pregnancy with cervical cancer, giant cervical fibroids, subuterine fibroids, etc.

^2^Malpresentation: fetal transverse position, first-born full-term single breech (estimated fetal birth weight > 3500 g) and foot first exposure

In the Uygur groups, there were 13 statistically significant independent variables, included maternal educational level, gravidity, parity, number of previous CD, scared uterus, prenatal anemia, placental abruption, placenta previa, stillbirth, emergency surgery, number of antenatal visits, intrapartum CD, general anesthesia. In the Han cohort, the 11 variables with *P* value < 0.05 in the univariate analyses were BMI, gravidity, parity, number of previous CD, scared uterus, diabetes, placenta previa, multiple pregnancy, macrosomia, number of antenatal visits, general anesthesia. Unadjusted and adjusted odds ratios for possible risk factors selected for multivariable analysis were showed in Tables 4 and 5 for Uygur and Han, respectively. For ethnic Uygur subgroup, placenta previa (aOR 11.18; 95% CI 3.09–40.45), general anesthesia (aOR 7.78; 95% CI 2.31–26.20; reference group = spinal anesthesia), prenatal anemia (aOR 4.84; 95% CI 2.44–9.60) and emergency surgery (aOR 4.22; 95% CI 1.95–9.13) were independently associated with PPH. For ethnic Han subgroup, placenta previa (aOR 20.08; 95% CI 6.35–63.46) and general anesthesia (aOR 5.70; 95% CI 1.89–17.26; reference group = spinal anesthesia) were also more likely to PPH. Other risk factors for ethnic Han were BMI (aOR 1.19; 95% CI 1.07–1.31) and multiple pregnancy (aOR 7.21; 95% CI 1.61–32.37). The Hosmer–Lemeshow test for the models was *P* value 0.87 (Uygur group) and 0.47 (Han group), indicating that the models had modest ability to discriminate patients with or without PPH.

**Table 4:** Identification of predictive factors of postpartum hemorrhage among Uygur subgroup

Risk factors	Unadjusted odds ratio^1^	Adjusted odds ratio^2^	*P* value^3^
Maternal educational level	0.53 (0.38–0.74)	0.53 (0.38–0.74)	0.63
Gravidity	2.85 (2.09–3.89)	1.33 (0.68–2.59)	0.41
Parity	3.71 (2.63–5.24)	1.67 (0.73–3.80)	0.22
Number of previous CD	2.84 (2.00–4.03)	1.38 (0.51–3.79)	0.53
Previous uterine scar	4.54 (2.79–7.39)	2.33 (0.59–9.19)	0.23
Prenatal anemia	6.80 (4.01–11.52)	**4.84 (2.44–9.60)**	**0.00**
Placental abruption	7.24 (2.30–22.76)	1.50 (0.28–8.15)	0.64
Placenta previa	17.64 (6.64–46.89)	**11.18 (3.09–40.45)**	**0.00**
Stillbirth	17.18 (2.12–139.16)	2.33 (0.21–25.70)	0.49
Emergency surgery	3.27 (1.92–5.56)	**4.22 (1.95–9.13)**	**0.00**
Number of antenatal visits	0.89 (0.83–0.96)	1.04 (0.95–1.14)	0.38
Intrapartum CD	0.37 (0.16–0.87)	1.08 (0.35–3.31)	0.89
General anesthesia	15.29 (6.17–37.90)	**7.78 (2.31–26.20)**	**0.00**

*PPH* postpartum hemorrhage, *CD* cesarean delivery

^1^Statistically significant associations in the univariable model are presented

^2^Statistically significant associations in the multivariable model are denoted by bold text and only variables with *P* value < 0.05 comes into multivariable model after univariable logistic regression

^3^The *P* value represents the statistical significance of all factors in the multivariable model

**Table 5:** Identification of predictive factors of postpartum hemorrhage among Han subgroup

Risk factors	Unadjusted odds ratio^1^	Adjusted odds ratio^2^	*P* value^3^
BMI (weight/height^2^)	1.13 (1.04–1.22)	**1.19 (1.07–1.31)**	**0.00**
Gravidity	1.76 (1.30–2.39)	1.17 (0.74–1.86)	0.50
Parity	2.09 (1.40–3.12)	0.95 (0.42–2.15)	0.90
Number of previous CD	2.59 (1.60–4.17)	1.16 (0.31–4.28)	0.83
Previous uterine scar	2.78 (1.63–4.71)	2.57 (0.63–10.51)	0.19
Diabets	2.90 (1.45–5.82)	1.88 (0.81–4.38)	0.14
Placenta previa	16.40 (6.09–44.19)	**20.08 (6.35–63.46)**	**0.00**
Multiple pregnancy	4.28 (1.26–14.62)	**7.21 (1.61–32.37)**	**0.01**
Macrosomia	1.94 (1.04–3.61)	1.43 (0.63–3.21)	0.39
Number of antenatal visits	0.89 (0.84–0.95)	0.94 (0.87–1.01)	0.08
General anesthesia	3.49 (1.45–8.39)	**5.70 (1.89–17.26)**	**0.00**

*PPH* postpartum hemorrhage, *BMI* body mass index, *CD* cesarean delivery

^1^Statistically significant associations in the univariable model are presented

^2^Statistically significant associations in the multivariable model are denoted by bold text and only variables with *P* value < 0.05 comes into multivariable model after univariable logistic regression

^3^The *P* value represents the statistical significance of all factors in the multivariable model

## Discussion

Our study confirmed that ethnic Uygur is a risk factor for PPH compared with ethnic Han in CD cohort. In different ethnic populations, general anesthesia and placenta previa were common risk factors for PPH and placenta previa was the factor with the highest risk. For women undergoing CD with general anesthesia, the Uygur were more likely to have PPH. Prenatal anemia and emergency surgery were risk factors for Uygur while BMI and multiple pregnancy were risk factors for Han.

PPH has been the leading cause of maternal mortality in China and worldwide [5, 25, 26]. While risk factors for PPH have been extensively studied, little is known regarding racial-ethnicity disparities in PPH, particularly in China. Among previous studies worldwide documenting whether racial-ethnicity disparities exist with regard to PPH, one study was conducted in vaginal birth [27] and the other three studies were not able to adequately adjust for potential confounding factors (e.g. maternal education level, age, BMI, macrosomia) [8, 9, 28]. Moreover, no study has analyzed such association in specific ethnic cohorts.

The causes of PPH include uterine contraction, genital tract trauma, placental factors, damage and abnormal blood coagulation. Various factors influence each other, and PPH is often the result of several factors. Though the uterine atony occurs in only 5% of labor, approximately 80% of PPH cases result from the uterine atony [6]. However, the uterine atony can take place secretively with no predictive factors. It may result from an inadequate response to endogenous and exogenous signals [8]. Many PPH cases often occur from uterine atony do not have identifiable antepartum risk factors [6]. Therefore, we should not only pay attention to the high-risk factors, but also monitor and intervene in women with hidden risk factors like ethnicities. Ethnic disparities in pregnant women's health and obstetric outcomes may mainly result from social–economical factors, including language barriers, healthcare resources, cultural barriers, and genetic differences. Ethnic-specific risk factors, such as multiple pregnancy and high BMI in ethnic Han and prenatal anemia and emergency surgery in Uygur, were found in our study.

Anesthesia is necessary for women undergoing CD and whether general anesthesia or spinal anesthesia is a risk factor for PPH is worth studying [24]. Previous studies have shown that, in general anesthesia, many drugs could suppress the contraction of animal and human uterine muscles, like intravenous general anesthetics which include propofol, midazolam, ketamine and opioids [29], volatile anesthetics which include sevoflurane and desflurane [30]. On the other hand, general anesthetics might suppress platelet function and hemostasis. Sevoflurane is demonstrated to alter bleeding time in a reversible and dose-related manner [31]. The intravenous anesthetic propofol may inhibit platelet aggregation [32]. Potential causes coming from the general anesthetics should be discussed. However, we need to notice that general anesthesia or intubation is a part of protocol for critical postpartum complications so the anesthesiologists may prefer to use general anesthesia for patients with obstetric complications. Previous study by Butwick et al. [24] has conducted the sensitivity analyses for the pre-labor cohort. They excluded the conditions that general anesthesia may be considered more often for women with obstetric complications including placenta previa or abnormal placentation. The results showed that the relationship between general anesthesia and severe PPH still persisted, albeit with wider confidence intervals. Nevertheless, the relationship between PPH and general anesthesia should be considered in cautions, and further studies are in need.

### Strengths and limitations

The large number of births in the Xinjiang Uygur Autonomous Region Maternal and Child Health Hospital accumulates sufficient cases undergoing CD and suffering from PPH to examine risk factors among different ethnic groups. We incorporated various aspects of covariates categorized as demographic characteristics, obstetric characteristics and comorbidities, fetal conditions and clinical management. Particularly, we were able to examine the role of anesthesia methods and emergency CD in the association between ethnicity and PPH, which were rarely addressed by previous studies. By robustly adjusting the role of comprehensive potential covariates of the association of ethnicity and PPH, we made valid conclusions.

Our study, however, had a number of limitations. The sample size of control group was small compared with 11,534 non-PPH subjects which we sampled from. Potential selection bias may affect the results, even though we used two matched factors to control the selection bias. Despite our efforts to adjust for a full range of candidate variables, some unmeasured factors, such as maternal socioeconomic status, prolonged third-stage labor [33], vertical incision, preeclampsia and uterine incision [34], were not captured in our analyses. We did not collect data for the parturients by vaginal delivery because the CD has potential risks related to numerous complications and it is a cause of PPH [5], besides, CD rate is elevating rapidly worldwide [35].

## Conclusion

The current study confirmed that maternal ethnicity is an independent risk factor for PPH and provided insights into the risk factors for PPH in different ethnicities. Hence, it may be important to stratify women by ethnicities for prevention, intervention, and treatment of the PPH. By augmenting access to health education, services and opportune perinatal care in the specific ethnic, the risk of PPH may reduce. Such analyses would require population-wide studies using nuanced clinical data. The epidemiological results will also be conducive to identify future research fields aimed at risk factors for PPH.

## Data Availability

The datasets used and/or analysed during the current study are available from the corresponding author on reasonable request.

## Abbreviations

PPH: Postpartum hemorrhage
CD: Cesarean delivery
ICD-9-CM codes: International Classification of Diseases 9th revision codes, Clinical Modification codes
ASA: American Society of Anesthesiologists
EBL: Estimated blood loss
RBC: Red blood cell
BMI: Body mass index
CDMR: Cesarean delivery on maternal request
ORs: Odds ratios
95% CI: 95% Confidence intervals
aOR: Adjusted odds ratio
VIF: Variance inflation factor
